# Androgen receptor and heat shock protein 27 co-regulate the malignant potential of molecular apocrine breast cancer

**DOI:** 10.1186/s13046-018-0762-y

**Published:** 2018-04-27

**Authors:** Xiaozhen Liu, Changyun Feng, Junjun Liu, Lu Cao, Guomin Xiang, Fang Liu, Shuling Wang, Jiao Jiao, Yun Niu

**Affiliations:** 1 Department of Breast Cancer Pathology and Research Laboratory, Tianjin Medical University Cancer Institute and Hospital, National Clinical Research Center of Cancer, Key Laboratory of Cancer Prevention and Therapy, Tianjin, Tianjin’s Clinical Research Center for Cancer, Tianjin, 300060 China; 2 Key Laboratory of Breast Cancer Prevention and Therapy, Tianjin Medical University, Ministry of Education, West Huanhu Road, Ti Yuan Bei, Hexi District, Tianjin, 300060 China; 3 Department of Maternal and Child Health Hospital of Linyi, Qinghe-South Road, Luozhuang District, Linyi, 276016 China

**Keywords:** Androgen receptor, Heat shock protein 27, Molecular apocrine, Phosphorylation

## Abstract

**Background:**

The most striking feature of molecular apocrine breast cancer (MABC) is the expression of androgen receptor (AR). We report here the mechanism of the AR in regulating the behavior of MABC.

**Methods:**

The MABC cell line, MDA-MB-453, and the nonMABC cell line, MCF7, were used in this study. The effect of dihydrotestosterone (DHT) and heat shock protein 27 (HSP27) on cell proliferation was quantified using the cell counter kit-8 (CCK8) and clonogenic assays in vitro and by a xenograft tumor model in vivo. The expression of the AR and HSP27 was analyzed using western blot, qPCR, and immunofluorescence assays. Complexes of the AR and HSP27 were detected by co-immunoprecipitation (Co-IP).

**Results:**

In MDA-MB-453 cells, DHT promoted cell proliferation and stimulated AR and HSP27 translocation from the cytoplasm to the nucleus, whereas, it inhibited MCF7 cell growth, and only the AR translocated into the nucleus. *HSP27* knock-down decreased the proliferative ability of MDA-MB-453 cells, which could be rescued by DHT, while HSP27 and DHT had synergistic effects on MCF7 cells. HSP27 phosphorylation was a prerequisite for AR translocation into the nucleus, especially phosphorylation on serine 82. In addition, DHT stimulated the tumorigenic and metastatic capacities of MDA-MB-453 cells, while *HSP27* knock-down decreased the rate of tumor formation and induced apoptosis in cells.

**Conclusions:**

The results suggest that HSP27 assists the AR in regulating the malignant behavior of MABC, and these findings might be helpful in the treatment of MABC.

**Electronic supplementary material:**

The online version of this article (10.1186/s13046-018-0762-y) contains supplementary material, which is available to authorized users.

## Background

As a member of the nuclear receptor family, the androgen receptor (AR) plays an important role in breast cancer, and has been identified as a biomarker for a specific molecular subtype of breast cancer. The AR gene expression profile can be used for further classifying receptor-negative tumors as molecular apocrine breast cancer (MABC) [1]. MDA-MB-453 breast cancer cells have been classified as molecular apocrine by gene profiling studies [2]. Our previous study has stated that patients with MABC develop distant metastases earlier and have poor prognosis [3]. As MABC is characterized by increased androgen signaling, the malignant potential of MABC may partly be because of the AR. A study has shown that when the AR in MDA-MB-453 breast cancer cells is knocked down, the cell colony formation rate is significantly decreased, which verifies the fact that the AR regulates the biological behavior of MABC [4]. Both ligand binding and translocation from the cytoplasm to the nucleus play important roles in the function of the AR [5]. However, the specific mechanism of cytoplasmic and nuclear translocation of the AR has not been clarified in MABC.

In the absence of ligand, the AR remains in a non-active state, which forms a protein complex with heat shock proteins (HSPs) and other co-chaperones in prostate cancer, while the AR becomes active when ligand binds, and it translocates to the nucleus as a transcriptional regulator [6]. Based on this result, HSPs play crucial roles in the process of AR activation. As a member of the molecular chaperones, HSP27 forms a chaperoning oligomer which can regulate multiple cellular survival and signaling pathways [6]. However, whether HSP27 combines with the AR during its translocation from the cytoplasm to the nucleus in MABC cells remains unclear. Furthermore, in the process of AR translocation to the nucleus, the rapid phosphorylation of HSP27 is regulated by ligand binding [6, 7]. There are three serine residues, serine 15, 78, and 82, reported as the sites of human HSP27 phosphorylation [8, 9]. However, which one of these residues plays an indispensable role for AR cytoplasmic and nuclear translocation still remains unknown.

To detect the specific mechanism of AR cytoplasmic and nuclear translocation in MABC cells, androgen and siRNAs specific for *HSP27* were used to analyze the location of the AR and HSP27 in vitro, and their effects on the tumorigenic capacity of MABC cells in vivo. The results of this study could determine the mechanism of the AR in regulating the malignant potential of MABC. Additionally, it aims to explore potential therapeutic targets for patients with MABC.

## Methods

### Cell lines and culture

Because the MDA-MB-453 cell line is classified as a model of MABC [2], and MCF7 cells as nonMABC cell line [10], we obtained them from American Type Culture Collection (ATCC, USA) for this study. The MDA-MB-453 cells were cultured in L15 medium (Gibco, USA), containing 10% fetal bovine serum (FBS, Gibco, USA) and 1% penicillin/streptomycin (Life Technologies, USA). MCF7 cells were cultured in DMEM medium (Gibco, USA) which contained 10% FBS and 1% penicillin/streptomycin. Both cell lines were incubated at 37 °C in 5% CO_2_.

### Plasmids and transfection

The *HSP27* siRNAs and control plasmids were constructed by Genechem (China). Three target sequences for the *HSP27* siRNAs were studied, which included siRNA#4892-1: 5′-CTGTGAGGACTGTGGATAA-3′, siRNA#4893-1: 5′-CCCAGCAAATCCCTCTCTA-3′ and siRNA#4894-2: 5′-GGCAAGTTCCAGGCATTT-3′.

The deletion of HSP27 phosphorylation sites (Ser15, Ser78 and Ser82; CS-I0586-Lv201-01, CS-I0586-Lv201-02, and CS-I0586-Lv201-03) were carried out by GeneCopoeia (China, Additional file 1). The plasmids were amplified in *E. coli*, and then extracted by using the Endotoxin-free Plasmid Size Kit (TIANGEN, China).

Cell transfections were performed as follows: firstly, cells were seeded in 6-well plates at a density of 1.0 × 10^4^ cells per well overnight. Subsequently, 2 μg of the constructed plasmids were added to MEM (Gibco, USA), respectively, and incubated for 5 min at 37 °C. Further, the FuGENE Transfection Reagent (Promega, USA) was added and mixed. After incubating for 15 min, the complex solution was added to the cells, and replaced with complete medium 8 h later. The reactions were incubated for 48 h.

### Quantitative real-time PCR (qPCR)

The RNAs used in this study were extracted using Trizol reagent (Takara, Japan). Reverse transcription was carried out using the SuperScript RT kit (Takara, Japan). qPCRs were performed according to the manufacturer’s protocol using the SYBR Green PCR kit (Toyobo, Japan). The transcript level of *GAPDH* was adopted as an internal control, and the primers used were as follows: *GAPDH*: 5′-GGAAGGTGAAGGTCGGAGTC-3′ and 5′-GTCTTCTGGGTGGCAGTGAT-3′; *AR*: 5′-GGAATTCCTGTGCATGAAA-3′ and 5′-CGAAGTTCATCAAAGAATT-3′; *HSP27*: 5′- GCGTGTCCCTGGATGTCAAC-3′ and 5′-TGTATTTCCGCGTGAAGCAC-3′. Each sample was assayed in triplicate.

### Western blot analysis

Total proteins were extracted with RIPA buffer (Thermo Scientific, USA) and 1 mM PMSF. Cytoplasmic and nuclear subcellular fractionation was performed according to the manufacturer’s instructions using the Nuclear and Cytoplasmic Isolation Kit (KeyGEN, China). All proteins were separated on 10% SDS-PAGE (Invitrogen, USA) gels, transferred onto PVDF membranes (Millipore, USA), and then blocked using 5% skim milk. The proteins were detected by incubating the following primary antibodies: anti-AR (AR441; Abcam, USA), anti-estrogen receptor (ER; D8H8; CST, USA), anti-progesterone receptor (PR; 6A1; CST, USA), anti-HSP27 (G3.1, Abcam, USA), anti-HSP27 (phospho S15) (EP2293Y, Abcam, USA), anti-HSP27 (phospho S78) (Y175, Abcam, USA), anti-HSP27 (phospho S82) (EPR7278, Abcam, USA), anti-β-actin (8H10D10, CST, USA), and anti-Histone H3 (D18C8, CST, USA) overnight; and incubated with horseradish peroxidase-labeled anti-rabbit or anti-mouse IgG, followed by detection using the ECL detection kit (Solarbio, China). Each sample was analyzed in triplicate.

### Co-immunoprecipitation(Co-IP) and western blot

Co-IP of the AR and HSP27 was carried out according to the manufacturer’s protocol using the Pierce Co-IP kit (Thermo Scientific, USA). Briefly, AR or HSP27 antibody (10 μL) was first incubated with the AminoLink Plus coupling resin. The antibody-coupled resin was incubated with protein lysates overnight. Subsequently, the resin was washed, followed by elution of the protein complexes, which bound to the AR or HSP27 antibody. Subsequently, the samples were detected by western blot using the HSP27 or AR antibody as described previously. Each sample was assayed in triplicate.

### Cell counter kit-8 (CCK8) cell proliferation and clonogenic assays

Cells (DHT treatment or *HSP27* knock-down) were suspended and seeded in 96-well plates at 5000 cells per well and incubated for 24 h. Further, 10 μL of CCK8 (KeyGEN, China) solution was added into each well on day 1, 2, 3, 4 and 5. After 1–4 h, the absorbance of each well was measured at 450 nm using a microplate reader. Clonogenic assays were carried out using 6-well plates with 1000 cells per well. After 15 days, cells were collected and stained with crystal violet, and then the number of cell colonies was counted. Each sample was assayed in triplicate.

### Immunofluorescence (IF) assay

Cells were seeded in 24-well plates. After 24 h, cells were fixed with 4% paraformaldehyde, permeabilized with 0.2% Triton X-100, blocked with bovine serum albumin, incubated with the primary antibodies and fluorescein-labeled secondary antibody, and then DAPI (Thermo Scientific, USA) was used to stain the nucleus. Images were visualized and analyzed using a fluorescence microscope. Each sample was analyzed in triplicate.

### In vivo experiments

Eighteen female BALB/c-nude mice (4–6 weeks old, 18–20 g) were purchased and randomly divided into three groups: MDA-MB-453 cells with DHT treatment, *HSP27* knock-down, and the control group. Cells (2 × 10^6^) were inoculated subcutaneously in the groin. Care and procedures of the mice were provided by the Institution of Animal Use and Care Committee of Tianjin Medical University Cancer Institute and Hospital. All mice were sacrificed until 55 days. Tumor volumes were calculated as previously reported [11].

All the tumors, livers, and lungs were paraffin-embedded and stained with hematoxylin-eosin (HE). HSP27 and Ki67 expression of mouse tumors were analyzed by immunohistochemistry as previously reported [3]. In order to detect apoptotic cells, the terminal deoxynucleotidyl transferase dUTP nick end labeling (TUNEL) assay was carried out according to the manufacturer’s instructions, and the kit was obtained from KeyGEN (China).

### Statistical analysis

Statistical analyses were performed using SPSS 19.0 software. The data were recorded as means ± standard deviation from at least three independent experiments, and analyzed by one-way ANOVAs and T-tests. *P* < 0.05 was considered as statistically significant.

## Results

### Determination of cells and DHT working conditions

**Fig. 1:**
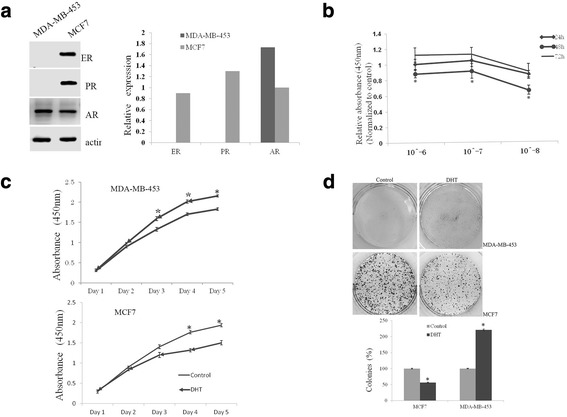
DHT affected the proliferation ability of breast cancer cells. Western blot analyzed the expression of ER, PR, and AR in MDA-MB-453 and MCF7 cells, and quantified by the bar graph (**a**). CCK8 assay determined the working conditions of DHT (**b**). The proliferation ability of MDA-MB-453 and MCF7 cells treated with or without DHT was measured by CCK8 (**c**) and clonogenic (**d**) assays. *P < 0.05

Two breast cancer cell lines were used in this study, MDA-MB-453 and MCF7, which are representative of MABC and nonMABC, respectively. Cells were tested by western blot to determine the expression levels of ER, PR, and AR. MDA-MB-453 cells demonstrated no detectable levels of ER or PR, but high levels of AR, while all of these proteins could be detected in MCF7 cells. However, the level of AR protein was 1.73-fold higher in MDA-MB-453 than in MCF7 cells (Fig. 1a).

To determine whether dihydrotestosterone (DHT) could affect the proliferation of breast cancer cells, MCF7 cells were cultured in 96-well plates in medium containing 10% dextran-coated charcoal-treated FBS (DCC-FBS) incubated with or without different concentrations of DHT (10^− 6^, 10^− 7^, and 10^− 8^ M) for 24, 48, or 72 h. CCK8 assays showed that the effects of DHT were dose and time dependent, as the growth inhibitory effects could work best at a concentration of 10^− 8^ M and simultaneously for 48 h (Fig. 1b). Therefore, a 10^− 8^ M concentration for 48 h was considered as the optimal condition for DHT in the following studies.

### Effects of androgen on MABC cell proliferation

The effect of DHT on MDA-MB-453 cell proliferation when exposed to DHT for 1–5 days was examined with the CCK8 assay, and the MCF7 cell line was used as a control. A significant anti-proliferative effect by DHT on MCF7 cells could be observed at the fourth day. However, a significant promotion of cell proliferation in MDA-MB-453 cells by DHT treatment was detected at the third day (Fig. 1c). Clonogenic assays also showed that the number of colonies formed by MDA-MB-453 cells treated with DHT increased dramatically compared to the control cells (221% vs. 100%, respectively), while it decreased in MCF7 cells with DHT treatment compared to the control cells (56% vs. 100%, respectively; Fig. 1d). Therefore, proliferation of MDA-MB-453 and MCF7 cells showed opposite responses to DHT treatment.

### Effects of androgen on AR and HSP27 expression and localization

**Fig. 2:**
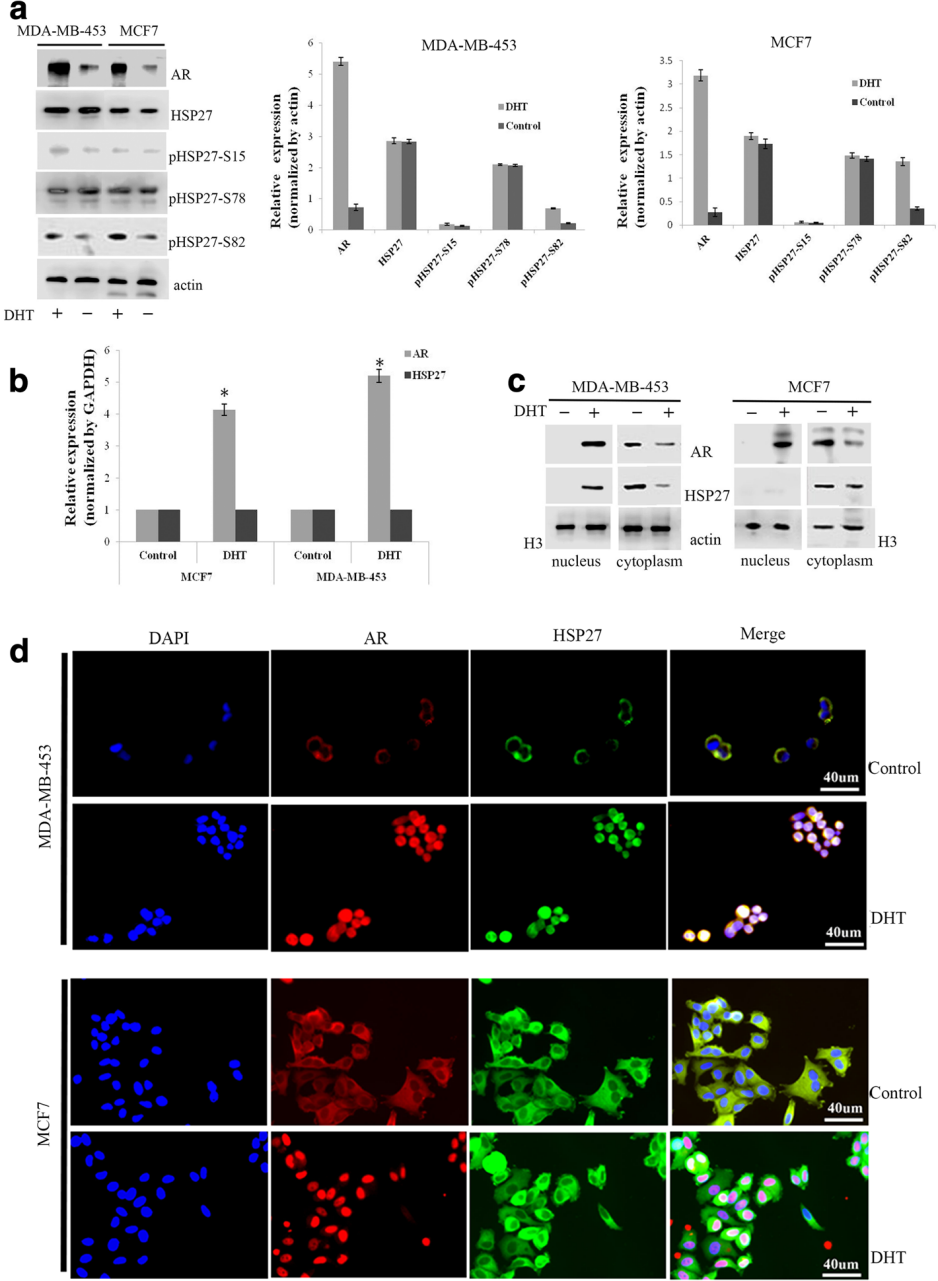
DHT affected the expression and location of AR and HSP27. The effect of DHT on the expression of AR, HSP27, and phosphorylated forms of HSP27 at serine 15, 78 and 82 was analyzed by western blot (**a**). The effect of DHT on the expression of AR and HSP27 mRNAs was measured by qPCR (**b**). The expression and location of AR and HSP27 in the cytoplasm and nucleus after MDA-MB-453 and MCF7 cells treated with or without DHT were analyzed by western blot (**c**) and immunofluorescence (**d**) assays. *P < 0.05

To determine whether androgen could affect the expression of the AR and HSP27, western blot was carried out, and the results showed a significant increase in the levels of AR protein in both MDA-MB-453 and MCF7 cells treated with DHT. However, DHT had no effect on the expression of HSP27. As HSP27 is phosphorylated on three serine residues (Ser15, Ser78, and Ser82), the phosphorylated forms of HSP27 after DHT treatment were analyzed. Interestingly, the expression of Ser82 significantly increased, while the others remained unchanged (Fig. 2a). The changes in *AR* and *HSP27* transcript levels in these two cell lines treated with or without DHT were consistent with the changes in protein level. However, the mRNA expression of *AR* was significantly increased while *HSP27*expression remained steady-state (Fig. 2b).

Furthermore, the protein levels of the AR and HSP27 in the cytoplasm and nucleus after subcellular fractionation were analyzed. In MDA-MB-453 cells, the levels of both the AR and HSP27 were significantly increased in the nucleus and decreased in the cytoplasm by DHT treatment, while only the level of the AR increased in the nucleus and decreased in the cytoplasm in MCF7 cells (Fig. 2c). In addition, IF assays illustrated that both the AR and HSP27 were localized in the nucleus of MDA-MB-453 cells with DHT treatment compared to the cytoplasm in control cells. However, only the AR was localized in the nucleus of MCF7 cells after DHT treatment (Fig. 2d).

### Mechanisms of DHT-induced AR and HSP27 cytoplasmic/nuclear translocation

**Fig. 3:**
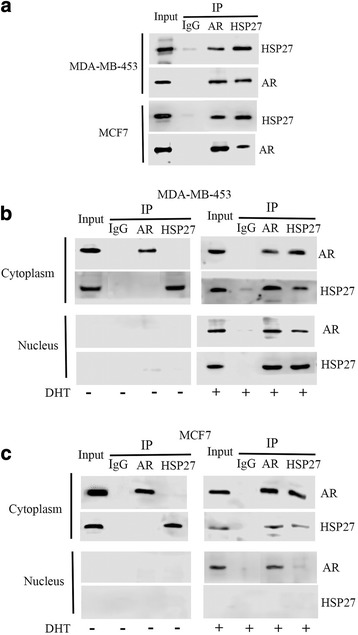
DHT induced the formation of AR-HSP27 complex. Co-IP for AR and HSP27 in the total proteins extracted from MDA-MB-453 and MCF7 cells treated with or without DHT (**a**). Co-IP for AR and HSP27 in the proteins extracted from the cytoplasm and the nucleus of MDA-MB-453 (**b**) and MCF7 (**c**) cells treated with or without DHT

To further confirm the mechanisms of DHT-induced AR and HSP27 translocation from the cytoplasm to the nucleus, the interaction between HSP27 and AR was analyzed by Co-IP methods. Firstly, total proteins were extracted from MDA-MB-453 and MCF7 cells treated with DHT. The results indicated that the AR could be detected in HSP27 immunoprecipitated complexes, and vice versa (Fig. 3a). Subsequently, a cytoplasmic and nuclear subcellular fractionation was performed. Analysis showed that only after DHT treatment, the AR-HSP27 complex could be detected in both the cytoplasm and nucleus of MDA-MB-453 cells (Fig. 3b), and in the cytoplasm of MCF7 cells (Fig. 3c).

### Effects of HSP27 on MABC cell proliferation

**Fig. 4:**
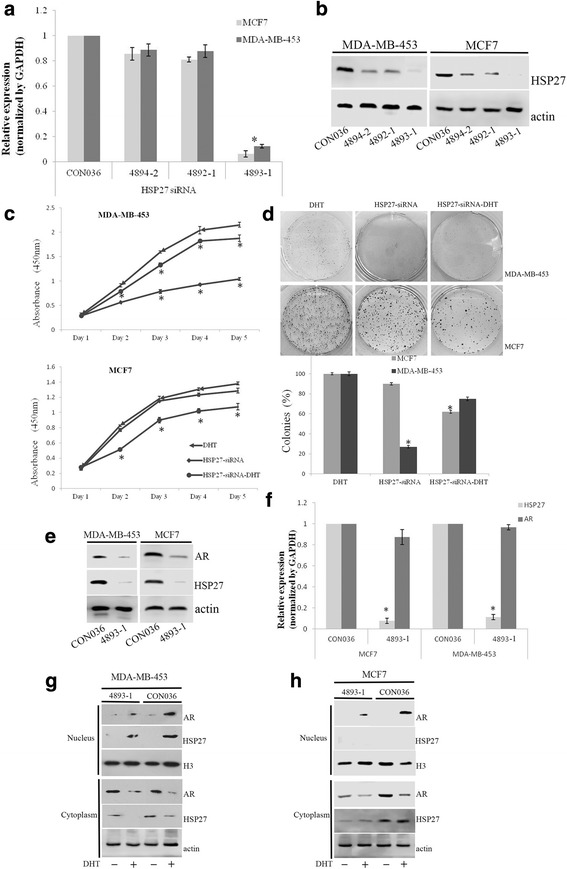
HSP27 affected the expression and location of AR. Verification of the siRNA specific for HSP27 at three sites (4894-2, 4893-1, 4892-1) was measured by qPCR (**a**) and western blot (**b**). The proliferation ability of *HSP27* knock-down cells treated with or without DHT was measured by CCK8 (**c**) and clonogenic (**d**) assays. The effect of *HSP27* knock-down on AR expression in MDA-MB-453 and MCF7 cells was analyzed by western blot (**e**) and qPCR (**f**). Western blot analyzed the level of AR expression in the cytoplasm and nucleus after cells transfected with siRNA specific for HSP27 in MDA-MB-453 (**g**) and MCF7 (**h**) cells. *P < 0.05

Cells transfected with siRNAs specific for *HSP27* were examined to explore the effect of HSP27 on AR expression. qPCR and western blot assays determined that *HSP27* siRNA#4893–1 could clearly decrease the expression of *HSP27* compared with the others (Fig. 4a and b). CCK8 assays showed that *HSP27* knock-down decreased the proliferative ability of MDA-MB-453 cells compared to DHT treated cells. This effect could partly be prevented by DHT treatment. The proliferative ability of MCF7 cells was also decreased by *HSP27* knock-down, which was similar to DHT treatment, and was more obvious when cells were treated with both *HSP27* siRNAs and DHT (Fig. 4c). Clonogenic assays indicated that for MDA-MB-453 cells, the number of colonies in the *HSP27* knock-down group showed a significant decrease compared to the DHT-treated group (27% vs. 100%, respectively). When cells knocked-down for *HSP27* were treated with DHT again, the number of colonies increased (75%). For MCF7 cells, *HSP27* knock-down decreased the number of colonies, almost to the same extent as with DHT (90% vs. 100%, respectively), and the number was much lower when cells were treated with both *HSP27* siRNAs and DHT (62%, Fig. 4d).

### Effects of HSP27 on AR expression and localization

The protein level of the AR in *HSP27* knock-down cells was detected by western blot, and the results showed that the down-regulation of HSP27 decreased the AR protein level in these cells (Fig. 4e). However, *HSP27* knock-down had no influence on the mRNA levels of *AR* (Fig. 4f). In addition, *HSP27* knock-down decreased the level of AR translocation into the nucleus in both MDA-MB-453 and MCF7 cells (Fig. 4g and h).

### The critical sites of HSP27 phosphorylation for AR cytoplasmic/nuclear translocation

**Fig. 5:**
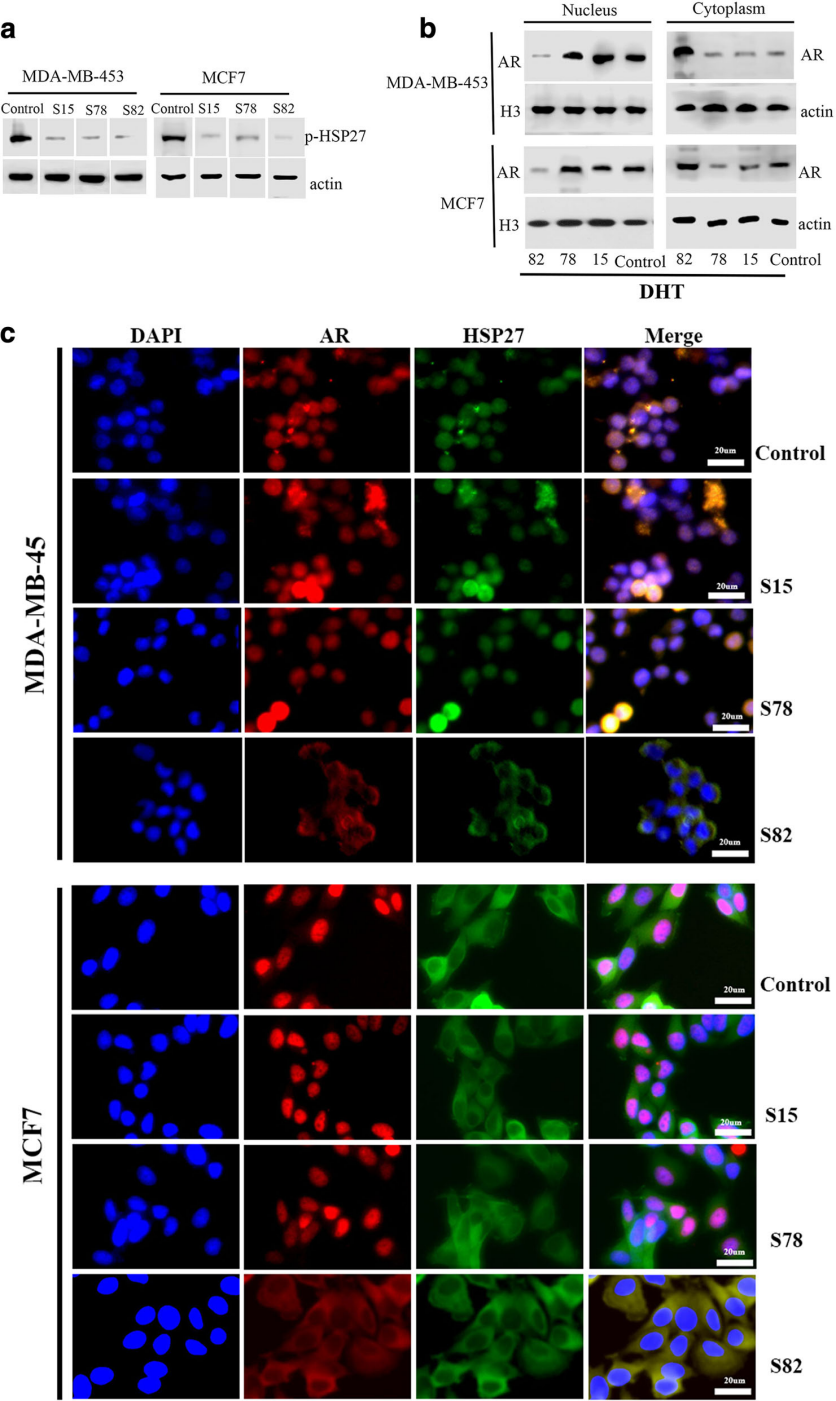
The critical sites of HSP27 phosphorylation for AR cytoplasmic/nuclear translocation. Verification of deleting the residues of HSP27 phosphorylation sites (serine 15, 78, and 82) was measured by western blot (**a**). The expression and location of AR and HSP27 in the cytoplasm and nucleus after HSP27 phosphorylation sites deleted and treated with DHT were analyzed by western blot (**b**) and immunofluorescence (**c**) assays

Cells transfected with HSP27 serine 15 deleted, HSP27 serine 78 deleted, and HSP27 serine 82 deleted constructs, respectively, were examined to confirm which site would play a critical role in AR cytoplasmic and nuclear translocation. Western blot was performed to verify the effects of the deletions (Fig. 5a). Cytoplasmic and nuclear subcellular fractionations from cells transfected with or without the specific plasmids and treated with DHT were analyzed. Compared to the control group, the level of the AR decreased in the nucleus and increased in the cytoplasm in cells transfected with HSP27 serine 82 deleted, while no significant differences were found in cells transfected with HSP27 serine 15 deleted or HSP27 serine 78 deleted and the control group (Fig. 5b). IF assays further verified that after DHT treatment, both the AR and HSP27 were localized to the cytoplasm of MDA-MB-453 and MCF7 cells transfected with HSP27 serine 82 deleted, compared to the nucleus of cells in the other three groups (Fig. 5c).

### Effects of androgen and HSP27 on the tumorigenic capacity of MABC cells

**Fig. 6:**
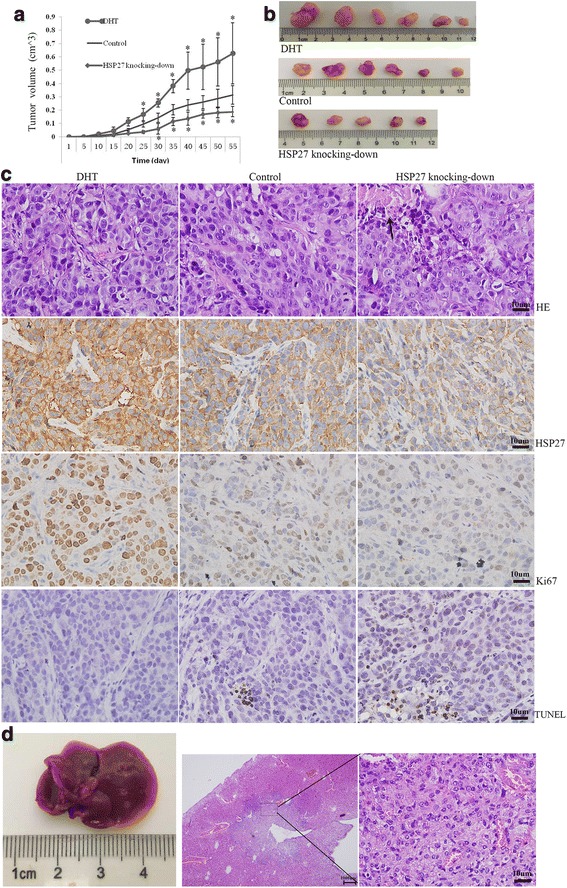
HSP27 and DHT affected the tumor formation and metastasis in vivo using MDA-MB-453 cells. The effect of DHT and *HSP27* knock-down on xenograft tumors growth and formation was described by tumor volume curve (**a**). Images of tumors removed from the mice (**b**). HE staining, immunohistochemistry staining for HSP27 and Ki67, and TUNEL staining were carried out on xenograft tumors (**c**). Representative images of liver with cancer cells invasion (**d**)

MDA-MB-453 cells (2 × 10^6^) were divided into three groups and injected into the mammary fat pad of mice. DHT treatment accelerated tumor growth significantly, while *HSP27* knock-down decreased the tumor formation rate compared to the control group (83.3% vs. 100%) and inhibited tumor volume significantly (Fig. 6a and b).

In sections with hematoxylin-eosin (HE) staining, no obvious difference was found in the tissue structure and cell morphology of xenograft tumors generated from these three groups; however, necrosis could be found in the *HSP27* knock-down group. Tumors generated from cells with DHT treatment expressed significantly higher Ki67 labeling, while tumors with *HSP27* knock-down expressed lower Ki67 compared to the control group. Apoptosis of cancer cells was evaluated by TUNEL staining. Tumors generated from the DHT treatment group showed the smallest proportion of TUNEL-positive cells, while the *HSP27* knock-down group showed the largest proportion compared to the control group (Fig. 6c). In addition, no metastases were found in the lungs among all these three groups; however, interestingly, there were two mice that developed liver metastases in the DHT treatment group (Fig. 6d).

## Discussion

Breast cancer is a heterogeneous tumor that can be reflected in different aspects, such as the classical histopathological features and the more modern molecular classification. MABC is defined as ER-negative, PR-negative, and AR-positive [12]; furthermore, with a poor prognosis by a series of clinical samples [3]. In this study, we further explored the mechanisms by which the AR regulates the malignancy of MABC*.* The results showed that HSP27 phosphorylation induced by androgen played a vital role in the process of AR translocation from the cytoplasm to the nucleus, which could affect the proliferation of tumor cells and tumorigenic capacity.

As a model of MABC, the proliferation of MDA-MB-453 cells is regulated by androgen in an AR-dependent manner [13–15]. In MABC cells, HSP27 was highly expressed and played a pivotal role in cell proliferation. In accordance with previous studies [2, 14, 16], we found that androgen could stimulate the proliferation of MDA-MB-453 cells. In contrast to the promotion effect on MABC cells, androgen has a predominantly inhibitory proliferative effect on MCF7 cells [17], a model of luminal breast cancer [10]. However, the effects of androgen on MDA-MB-453 cells could be partly rescued by down-regulation of HSP27, suggesting that knocking-down *HSP27* could reduce the androgen promotion effect on MDA-MB-453 cells. Additionally, HSP27 can decrease the toxic effects of oxidized proteins and reduce reactive oxygen species, which results in inhibiting cancer cell apoptosis [18–20]. These results suggest that androgen and HSP27 may interact with each other and co-regulate the proliferative ability of MABC cells.

HSP27 is a member of the HSPs family and has been stated to regulate the functions of the AR, such as AR cytoplasmic/nuclear translocation and AR transactivation [6, 21]. Studies confirm that gain and loss of function of HSP27 are strongly related to the expression of the AR in myogenic cells and prostate cancer cells [22, 23]. We also found that *HSP27* knock-down significantly decreased the level of AR protein, but no difference could be found in the level of AR mRNA, which suggests that HSP27 might only regulate the protein translation of AR in MABC cells. However, Stope et al. [23] stated that the decrease in the AR protein level is paralleled with a down-regulation in AR mRNA levels. This difference might be owing to the different cancer cells used for the research; however, the related mechanism should be further analyzed.

In malignant tumor cells, the expression of HSP27 is obviously high [24, 25]. The main mechanism by which the AR and HSP27 regulate the malignant behavior of MABC may rely on the the androgen-triggered phosphorylation of HSP27 that accompanies the AR tanslocation from the cytoplasm to the nucleus where the AR interacts with androgen response elements to promote its genomic activity. HSP27 is directly phosphorylated at three serine residues via the p38 mitogen-activated protein kinase (MAPK) pathway, which affects its subcellular distribution [26–28]. The phosphorylation sites at serine 78 and 82 are identified as the predominant residues of HSP27, and serine 78 can be substituted by asparagine in some animals, but serine 82 is conserved throughout the animals [9]. In MABC cells, deleting residue serine 82 of the HSP27 phosphorylation sites induced a significant decrease in the expression levels of AR in the nucleus compared to the control group, which indicated that HSP27 phosphorylation at serine 82 might be the critical site for AR cytoplasmic and nuclear translocation.

Several studies have confirmed that the positive expression of AR is significantly associated with poor survival, and increased mortality in AR-positive triple negative breast cancer (TNBC) [3, 29–31]. HSP27 is reported to be associated with a decreased survival in breast cancer [32]. In accordance with clinical research, we found that DHT treatment increased xenograft tumor volume and distant metastasis, while HSP27 knock-down inhibited tumor growth greatly, which indicated that the AR and HSP27 might interact with each other and co-influence the development of MABC. Studies have stated that AR antagonists are able to induce breast cancer cell apoptosis and decrease tumor proliferation significantly in TNBC cell lines [33, 34]. In addition, the HSP second generation antisense oligonucleotide targeting HSP27 can increase drug efficacy in pancreatic and prostate cancer xenograft models [35–37]. Based on these results, although no valid endocrine therapy is suggested for MABC, the combination of AR antagonists and HSP27 inhibitors could be a potential therapeutic regimen. Of course, more xenograft models and clinical trials should be carried out to confirm this hypothesis.

## Conclusions

In conclusion, this study confirmed that activation of AR and HSP27 by androgen could increase the proliferative ability of MABC cells and the growth of xenograft tumors. HSP27 phosphorylation on residue serine 82, induced by androgen, played a critical role in the process of AR translocation from the cytoplasm to the nucleus. The AR and HSP27 could form a protein complex, which was the main factor in AR regulating the malignancy behavior of tumor cells, and could present a new therapeutic regimen in clinical therapies of MABC.

## Additional file

Additional file 1: The sequence of the deleted HSP27 phosphorylation sites. (DOCX 13 kb)

## Abbreviations

AR: Androgen receptor
CCK8: Cell counter kit-8
Co-IP: Co-immunoprecipitation
DCC-FBS: Dextran-coated charcoal-treated FBS
DHT: Dihydrotestosterone
ER: Estrogen receptor
HE: Hematoxylin-eosin
HSPs: Heat shock proteins
IF: Immunofluorescence
MABC: Molecular apocrine breast cancer
MAPK: Mitogen-activated protein kinase
PR: Progesterone receptor
TNBC: Triple negative breast cancer
TUNEL: Terminal deoxynucleotidyl transferase dUTP nick end labeling
