# Supplementary material for: Androgen receptor and heat shock protein 27 co-regulate the malignant potential of molecular apocrine breast cancer
Source: J Exp Clin Cancer Res. 2018 Apr 27;37:90. doi: 10.1186/s13046-018-0762-y (PMC5921986; doi:10.1186/s13046-018-0762-y)
Supplement: Supplementary file 1 — The sequence of the deleted HSP27 phosphorylation sites. (DOCX 13 kb) [file 13046_2018_762_MOESM1_ESM.docx]

The deletion of HSP27 phosphorylation sites were carried out by GeneCopoeia (CS-I0586-Lv201-01, CS-I0586-Lv201-02, and CS-I0586-Lv201-03, China) in this study. The EX-I0586-Lv201 ORF sequence was as follows:

ATGACCGAGCGCCGCGTCCCCTTCTCGCTCCTGCGGGGCCCCAGC(deleted)TGGGACCCCTTCCGCGACTGGTACCCGCATAGCCG

CCTCTTCGACCAGGCCTTCGGGCTGCCCCGGCTGCCGGAGGAGTGGTCGCAGTGGTTAGGCGGCAGCAGCTGGCCAGGCT

ACGTGCGCCCCCTGCCCCCCGCCGCCATCGAGAGCCCCGCAGTGGCCGCGCCCGCCTACAGCCGCGCGCTCAGC(deleted)CGGCAA

CTCAGC(deleted)AGCGGGGTCTCGGAGATCCGGCACACTGCGGACCGCTGGCGCGTGTCCCTGGATGTCAACCACTTCGCCCCGGA

CGAGCTGACGGTCAAGACCAAGGATGGCGTGGTGGAGATCACCGGCAAGCACGAGGAGCGGCAGGACGAGCATGGCTACA

TCTCCCGGTGCTTCACGCGGAAATACACGCTGCCCCCCGGTGTGGACCCCACCCAAGTTTCCTCCTCCCTGTCCCCTGAG

GGCACACTGACCGTGGAGGCCCCCATGCCCAAGCTAGCCACGCAGTCCAACGAGATCACCATCCCAGTCACCTTCGAGTC

GCGGGCCCAGCTTGGGGGCCCAGAAGCTGCAAAATCCGATGAGACTGCCGCCAAG

The bases of the yellow label were the deleted serine (HSP27 phosphorylation sites: Ser15, Ser78 and Ser82).
